# Re-establishment of species from synonymies based on DNA barcoding and phylogenetic analysis using *Diplopterygium simulans* (Gleicheniaceae) as an example

**DOI:** 10.1371/journal.pone.0164604

**Published:** 2017-03-15

**Authors:** Jiang-Ping Shu, Hui Shang, Dongmei Jin, Hong-Jin Wei, Xi-Le Zhou, Hong-Mei Liu, Yu-Feng Gu, Ying Wang, Fa-Guo Wang, Hui Shen, Rui Zhang, Bayu Adjie, Yue-Hong Yan

**Affiliations:** 1 Shanghai Chenshan Plant Science Research Center, Chinese Academy of Sciences, Shanghai, China; 2 College of Life and Environmental Sciences, Shanghai Normal University, Shanghai, China; 3 Shenzhen Fairylake Botanical Garden, Chinese Academy of Sciences, Shenzhen, China; 4 South China Botanical Garden, Chinese Academy of Sciences, Guangzhou, China; 5 Bali Botanic Garden, Indonesian Institute of Science, Bali, Indonesia; Chinese Academy of Medical Sciences and Peking Union Medical College, CHINA

## Abstract

Because synonymy treatment traditionally relies on morphological judgments, it usually causes many problems in species delimitation and in the biodiversity catalogue. For example, *Diplopterygium simulans*, which belongs to the Gleicheniaceae family, has been considered to be synonymous with *D*. *glaucum* or *D*. *giganteum* based mainly on the morphology of its pinna rachis and blade. In the absence of molecular evidence, these revisions remain doubtful. DNA barcoding, which is considered to be a powerful method for species-level identification, was employed to assess the genetic distance among 9 members of the *Diplopterygium* genus. The results indicate that *D*. *simulans* is an independent species rather than a synonymy of *D*. *glaucum* or *D*. *giganteum*. Moreover, phylogenetic analysis uncovered the sisterhood of *D*. *simulans* and *D*. *cantonense*, which is supported by their geographical distributions and morphological traits. Incorrect synonymy treatment is prevalent in the characterization of biological diversity, and our study proposes a convenient and effective method for validating synonym treatments and discovering cryptic species.

## Introduction

How many species exist in a taxon is an intrinsically interesting question [1–5]. The description of new taxa and synonymy treatments should be the main approaches to answering this question [6]. With the development of molecular biology technology, an increasing number of species have been discovered based on molecular data analysis. However, identifying which species characterizations are good by screening synonymies and publications is difficult; phylogenetic reconstruction and DNA barcoding are considered to be good approaches [7].

Any errors in the determination of species units can lead to more serious errors in phylogenetic analyses that use species as the basic unit of analysis [8,9] and may cause many “good” species to possess highly conserved morphological features, leading to the commonly used term “cryptic species” [10]. Moreover, inaccurate assessment of species delimitation precludes the accurate inference of historical evolutionary processes [11]. Scientists have developed many effective methods to address this challenge [9,11,12]. Morphological data are the fundamental evidence used for species identification, and the majority of recognized species presumably have been delimited and described based on morphological differences [8]. DNA barcoding is a powerful method that is used to identify species and to draw attention to overlooked and new species to identify candidate exemplar taxa for comprehensive phylogenetic studies [13]. Thus, this method is widely used [14,15]. Here, we combined these two methods to identify species. Recently, many new species have been discovered based on these methods [16–18], but few synonymies were re-recognized [19].

*Diplopterygium* (Diels) Nakai (Gleicheniaceae) is an ancient lineage of leptosporangiate ferns. Its pioneer fossil has been dated to the Carboniferous, but the extant taxa of the genus appear to have diverged during the Early Cretaceous (111–140 Ma) [20]. The plants of the genus have important ecological roles [21,22]; as a ground cover layer, they hinder the absorption of litter into the earth’s surface, heavily reduce surface light, prevent soil evaporation, compete with tree seeds and affect the survival of dominant species and forest regeneration to a large extent [23]. However, the systematics and classification research for this genus has been highly controversial. Nakai (1950) conducted a preparatory study of all of the Gleichenious plants that have been described in the world, and he recognized 15 species in the genus; however, he admitted that some species and references needed to be revised [24]. Ching et al. (1959) recognized 17 species in China [25] by using *Hicriopteris* C. Presl, which was revised as *Diplopterygium* by Zhang [26]. Flora of China (English edition) recorded 9 species in China and approximately 20 species in the world [27]. A total of 132 legal names were published in the genus *Diplopterygium*, but only 22 names have been accepted until now. The naming of species in the *Diplopterygium* genus has been carried out many times based on morphological traits. However, the number of species in the genus remains uncertain.

Different taxonomists have conducted different treatments on different species of *Diplopterygium*. For example, the species *Diplopterygium simulans* (Ching) Ching ex X. C. Zhang was considered to be endemic to Hainan, similar to *Diplopterygium glaucum* (Thunberg ex Hoottuyn) Nakai [28,29]. In 2006, Wu treated *D*. *simulans* as a synonymy of *D*. *glaucum* [30]. Jin and Ding treated *D*. *simulans* as the synonymy of *Diplopterygium giganteun* (Wallich ex Hooker) Nakai in 2008 and 2013, respectively [27,31]. We found some differences and transitional traits among these species, such as the absence or presence of a narrow wing on the pinna rachis and the indumentum at the back of the blade. To examine the synonymy treatment of *Diplopterygium*, we collected fork fern species from China and neighboring areas and focused on the type locality of unaccepted species. First, we identified the specimens of the genus by using morphological features and then by using three genome regions (*rbcL*, *matK* and *trnL-F*) as barcodes to identify the species again; finally, we used five plastid genome regions, including coding and non-coding regions, to reconstruct the phylogenetic relationship of certain species.

## Materials and methods

### Ethics statement

In this study, we were not required any special permits, because our collection in the mainland of China was approved by the local departments and the Shanghai Chenshan Plant Science Research Center (Chinese Academy of Sciences), and the materials from Taiwan (China) and Bali (Indonesia) were provided by the collaborators. Moreover, all of the species we collected for this research are commonly found in the tropical and subtropical regions of the world, and none of them are endangered or protected species.

### Sample collection

A total of 65 accession samples were collected from 11 provinces in mainland China, Hainan Island, Taiwan Island, and Bali Island, Indonesia. Together, these samples represent 9 extant species of all 17 species of *Diplopterygium*. Two outgroup taxa belonging to two other genera (*Dicranopteris* Bernhardi and *Sticherus* C. Presl) were collected in China. The voucher specimens of all materials are kept in the Shanghai Chenshan Herbarium (CSH) and the Herbarium of the Shenzhen Fairylake Botanical Garden (SZG). Information about the specimens is shown in **S1 Table**.

### Morphological characters and geographical distribution

We tested six morphological characters of *D*. *simulans*, *D*. *giganteum*, *D*. *glaucum* and *Diplopterygium cantonense* (Ching) Nakai including the lobe width, tilt angle of the lobe, the number of lobe pairs, pinnule length, lobe length and the number of venation pairs. For each character, we measured more than eight specimens, and each sample was measured three times on the middle of different pinnule to obtain an average. Tukey’s HSD test was used to test for significant difference. The pictures of scale were obtained using the stereomicroscope (Nikon SMZ-1500, Japan) connected to a computer, and the pictures of the pinna were taken with a Digital Single Lens Reflex Camera (Nikon D90, Japan). Maps of the geographical distribution of the species were based on information about the specimens.

### DNA extraction, polymerase chain reaction, and sequencing

Test samples were sterilized with 75% ethanol, washed with distilled water, and then dried with silica. Each sample (20 mg) was ground to fine powder. The total DNA was extracted using the DNA secure Plant Kit (TIANGEN Corporation) according to the manufacturer’s instructions. The primers used for polymerase chain reaction (PCR) amplification are shown in **S2 Table**, and the amplification reaction was carried out in an Eppendorf gradient PCR amplification system. PCR amplification of four genomic regions (*rbcL*, *atpB*, *rps4*, and *trnL-F*) was performed in 20 μL volumes containing 10 μL of 2×*Taq* PCR MasterMix (TIANGEN). The volume of each primer was 0.25 μL. The volume of the primers for the *trnL-F* region was 0.6 μL; and the volume of the DNA template was 1 μL. ddH_2_O (TIANGEN) was added to the samples until they reached volume of 20 μL. However, PCR amplification of the *matK* region was performed in a volume of 30 μL containing 15 μL of 2×*Taq* PCR MasterMix, 1.2 μL of each primer, 9.6 μL of ddH_2_O and 3 μL of the DNA template. The reaction conditions for the amplifications of all the DNA regions are shown in **S3 Table**. The three steps of PCR thermocycling (denaturation, annealing, and extension) were conducted for 35 cycles, and another two steps were conducted for 1 cycle. Sequencing reactions were set up to obtain both the forward and reverse sequences, ethanol-precipitated, re-solubilized, and then sequenced on an ABI 3730xl DNA Analyzer (Applied Biosystems, Foster City, California, USA).

### Data analysis

Contig assembly and the generation of consensus sequences were performed using SeqMan v7.1.0 (DNASTAR, USA). The sequences used for DNA barcoding and phylogenetic analysis were aligned using BioEdit v7.2.0 [32], and the genetic distances were computed between two intraspecific or interspecific sequences using MEGA v6.06 [33] with the Kimura 2-Parameter (K2P) model [34]; the gaps and/or missing data were partially deleted (95%), and the other parameters were the default settings. The DNA barcoding gaps were determined to evaluate the distributions of intraspecific and interspecific divergences at the loci. The neighbor-joining (NJ) tree for the barcodes was constructed by MEGA v6.06 using a data matrix composed of three genome regions (*rbcL*, *matK* and *trnL-F*), and 1,000 bootstrap replicates were performed.

Phylogenetic trees were constructed using the maximum parsimony (MP) and maximum likelihood (ML) methods. MP analysis was performed using PAUP 4.0b10 [35]; gaps were treated as missing data and heuristic search options with 1,000 random replications of stepwise data addition and TBR swapping and Multrees on no-tree limit were used. Bootstrap analysis was performed with 1,000 replicates to evaluate the internal support with the addition of 1 random taxon replicate; all optimal trees were saved at each step [36]. The optimal model of molecular evolution was determined by the Akaike Information Criterion using Modeltest v3.7 [37,38]. An ML tree was constructed using PhyML v3.0 [39], and a GTR+I+G model was used. The proportion of invariant sites and state frequencies were estimated by the program. The genthreshfortopoterm option was set to 20,000, whereas the other settings were the default ones. To calculate the bootstrap support (BS) values for the ML tree, 1,000 replicates were carried out using the same criteria [40].

## Results

### DNA barcoding indicates that *D*. *simulans* is an independent species

**Fig 1:**
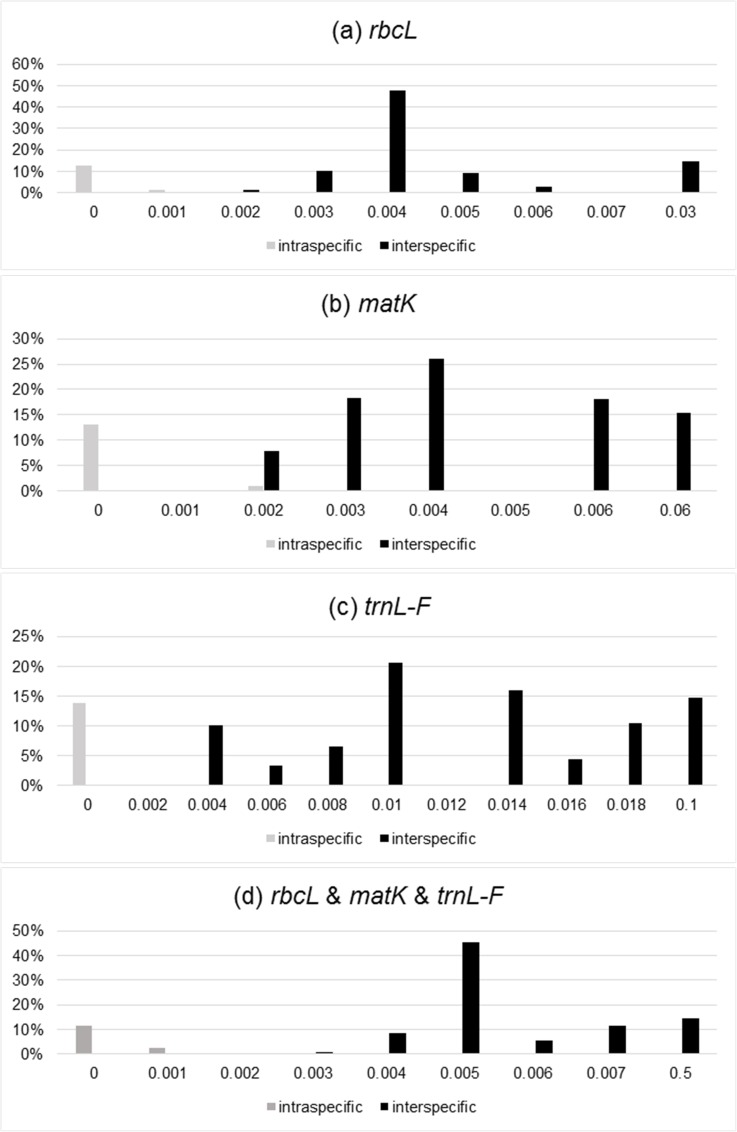
DNA barcoding Gap. Distribution of interspecific and intraspecific variation: (a) *rbcL*, (b) *matK*, (c) *trnL-F*, (d) *rbcL* & *matK* & *trnL-F*. The x-axis is the genetic distance and the y-axis is the frequency of the genetic distance.

**Fig 2:**
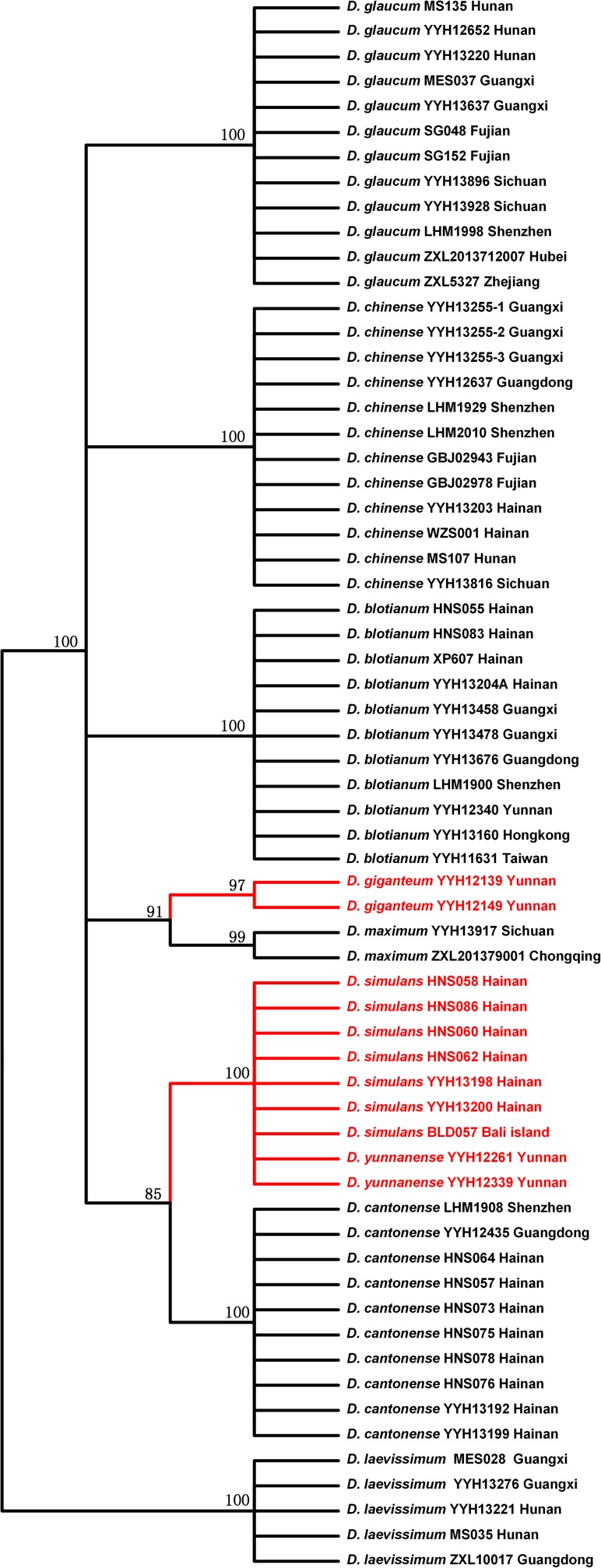
The Neighbor-joining Tree constructed from three chloroplast loci (*rbcL*, *matK* and *trnL-F*). The percentage of replicate trees in which the associated taxa clustered together in 1,000 bootstraps is shown next to the branches; values less than 70% were omitted. The red lines and words indicate one species (*D*. *giganteum*) based on the Flora of China (English edition, 2013).

We tested three DNA barcodes (*rbcL*, *matK*, and *trnL-F*) to identify the species in this genus. The PCR amplification rate of the three sequences from the *Diplopterygium* was 100%, and the sequencing success rate was 100%. We estimated the genetic divergences of 63 samples without two outgroups, and the distribution of intraspecific and interspecific variation is shown in **Fig 1**. The genetic distance between the intraspecies and interspecies is clear. This result showed that the markers *rbcL*, *matK* and *trnL-F* could be effective DNA barcodes for the genus, and the combination of these three loci provides a robust analysis. The NJ tree based on the combination of *rbcL*, *matK*, and *trnL-F* is shown in **Fig 2**. Based on the results, the species were divided into 9 groups, and each species that had been identified by morphological traits was gathered into a single monophyletic clade with robust BS (>70%). *D*. *simulans* was not grouped with *D*. *giganteum*.

### Phylogenetic analysis indicates that *D*. *simulans* is the sister of *D*. *cantonense*

**Fig 3:**
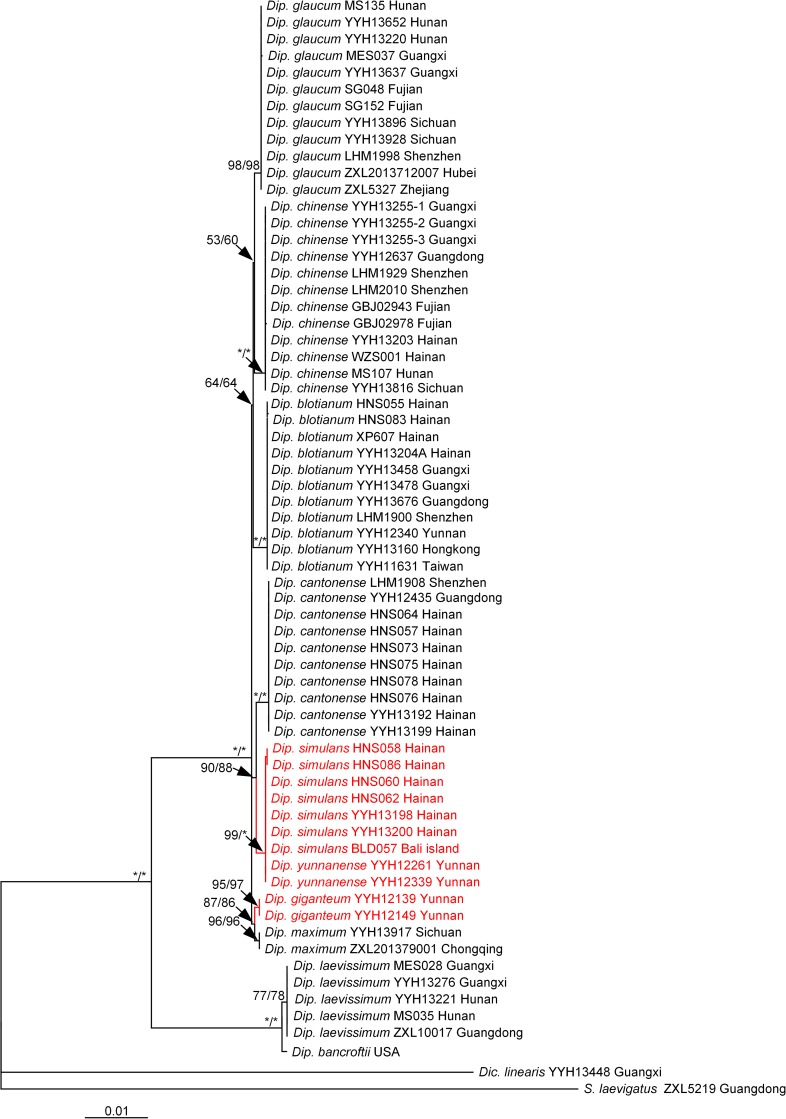
The phylogenetic tree constructed by MP and ML from five chloroplast loci (*rbcL*, *trnL-F*, *matK*, *atpB* and *rps4*). The red lines and words indicates one species (*D*. *giganteum*) based on the Flora of China (English edition, 2013). The symbol “*” indicates that BS = 100.

We used five loci (*rbcL*, *matK*, *trnL-F*, *atpB*, and *rps4*) to reconstruct the phylogenetic relationship in the genus. The results showed that *D*. *simulans* was the sister of *D*. *cantonense* **(Fig 3)**. The phylogenetic analysis strongly supported the monophyly of the *Diplopterygium* clade (BS = 100/100), and *Diplopterygium laevissimun* (H. Christ) Nakai together with *Diplopterygium bancroftii* (Hook.) A. R. Sm were shown to be the basal taxa of the genus. *D*. *giganteum*, *Diplopterygium maximum* (Ching) Ching & H. S. Kung formed one group, but their relationship could not clearly be inferred due to a lack of material.

### Geographical distribution and morphological differences

**Fig 4:**
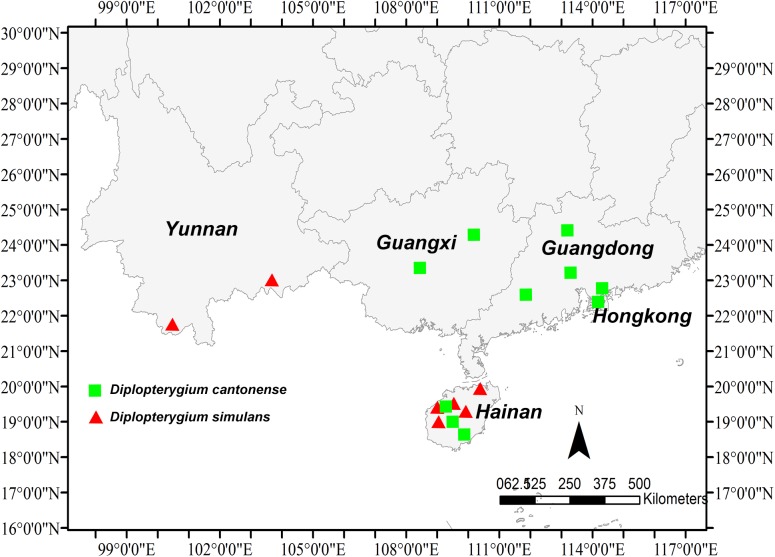
The geographical distributions of *D*. *cantonense* and *D*. *simulans*. The distributions of *D*. *cantonense* and *D*. *simulans* are the same. In addition to Hainan, *D*. *simulans* is distributed in Yunnan.

**Fig 5:**
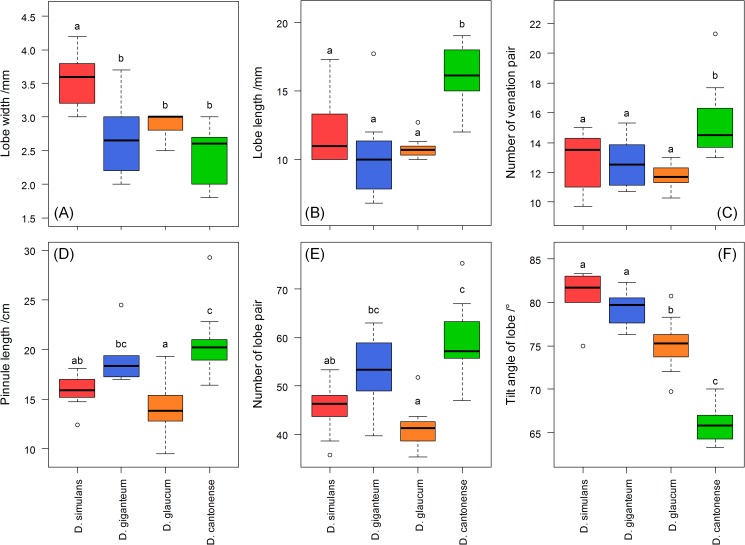
The differences in six morphological characters among four species (*D*. *simulans*. *D*. *giganteum*, *D*. *glaucum* and *D*. *cantonense*). The lower case letters (a, b, c) above the pillars are used to indicate significant differences; different letters indicate that the difference was significant; there were no significant differences between species when marked with the same letters.

**Fig 6:**
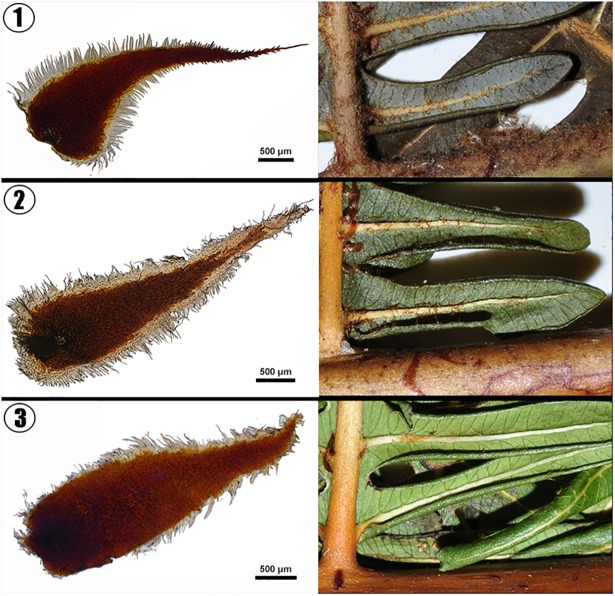
The forms of the squama and pinna for three species (*D*. *giganteum*, *D*. *simulans* and *D*. *cantonense*). 1. The squama and pinna of *D*. *giganteum*. 2. The squama and pinna of *D*. *simulans*. 3. The squama and pinna of *D*. *cantonense*. The abaxial side of the pinnule axis of *D*. *giganteum* has brown squama and a large amount of stellate hairs, which were very rarely observed in the other two species.

The geographical distributions for *D*. *cantonense* and *D*. *simulans* were obtained based on the information of the specimens. We found that these two species share the same range on Hainan Island. *D*. *cantonense* is also found in Guangdong and Guangxi, whereas *D*. *simulans* is found in Yunnan **(Fig 4)**. We measured six morphological characters for *D*. *simulans*, *D*. *giganteum*, *D*.*glaucum* and *D*. *cantonense D*. *cantonense* and *D*. *simulans* and then used Tukey’s HSD Test to test for significant of differences for all of the characters. The lobe width of *D*. *simulans* was significantly different than the other species, although there are no significant differences between the other species **(Fig 5A)**. There were no significant differences in the lobe lengths and the number of venation pairs among *D*. *simulans*, *D*. *giganteum* and *D*. *glaucum* **(Fig 5B and 5C)**. There were also no significant differences in pinnule length and the number of lobe pairs between *D*. *simulans* and *D*. *giganteum* or *D*. *glaucum* **(Fig 5D and 5E)**. The tilt angle of the lobe was significantly different in *D*. *simulans* compared with the other species except for *D*. *giganteum* **(Fig 5F)**. We took pictures of the scale and pinna for *D*. *simulans*, *D*. *giganteum* and *D*. *cantonense*. The abaxial side of the pinnule axis of *D*. *giganteum* has brown squama and a large amount of stellate hairs, which were very rarely found in the other two species **(Fig 6)**.

## Discussion

### Significance of rechecking synonymy

Paton et al. (2008) found a consistent percentage of synonymies within each family, considering the rate of synonymy, they estimated that 581843 synonymies exist in the flora [41]. Previous taxonomical revisions were based on the subjective judgment morphological characters. Thus, rechecking the revisions based on molecular biology is necessary. In recent years, many new species were identified by using the methods of molecular biology [16,18] and a few studies reanalyzed the synonymies. Liu et al. (2013) reinstated *Arthropteris guinanensis* H.G. Zhou & Y.Y. Huang as an independent species based on molecular data [19]; Li and Yao (2015) reinstated *Bridelia fordii* Hemsl, which is often treated as a synonym of *Bridelia retusa* (Linnaeus) A. Jussieu, as an independent species based on morphological and molecular data [42]; Chantarasuwan et al. (2015) reinstated *Ficus wightiana* Wall as an independent species based on molecular data, morphology, and leaf anatomy [43]. However, many species were not reexamined after being determined to be synonymies.

In our study, *D*. *simulans* was treated as the synonymy of *D*. *glaucum* [30] and *D*. *giganteum* [27] because of their similar morphological charaters **(Fig 5)**. However, molecular data showed that *D*. *simulans* was the sister species of *D*. *cantonense* **(Fig 3)**. These two species have a narrow distribution in China and are distributed sporadically in the tropics **(Fig 4)**. Several traits were found to differ between the two species (*D*. *simulans* and *D*. *giganteum*). For instance, the leaf axis of *D*. *simulans* has an obvious narrow wing, which does not occur in *D*. *giganteum*. In addition, the abaxial sides of the pinnule axes of *D*. *simulans* and *D*. *cantonense* have chaff-shaped dark brown squama and a few stellate hairs, which occurred less in *D*. *giganteum* **(Fig 6)**. Furthermore, there were significant differences between *D*. *simulans* and the other three species **(Fig 5A)**. According to the DNA barcoding results, a clear gap exists between the intraspecific and interspecific distances **(Fig 1)**, and the NJ tree showed that these three species were independently monophyletic **(Fig 2)**. Thus, in the present study, we determined that *D*. *simulans* is an independent species and not a synonym of *D*. *giganteum*.

Many revisions of synonymies are based only on a few morphological judgments with little evidence, and many species have disappeared from the taxonomic checklist. Thus, new species should be discovered to recheck those disposed synonymies based on molecular phylogeny and DNA barcoding after extensive sampling from the type locality.

### Species delimitation

Accurate species delimitation continues to pose a major challenge for systematics and evolutionary research [11,44]. In this study, we identified species by a combination of morphological characters and DNA barcodes. First, the majority of recognized species have presumably been delimited and described based on morphological differences. Species are delimited based on one or more qualitative or quantitative morphological characters that show no overlap with other species. This criterion is traditional but makes sense biologically. If two species are consistently distinguished by one or more diagnostic morphological differences, then there is presumably no gene flow between them (given some assumptions, such as the idea that each morphological difference has a genetic basis) [8].

Since its conception [12], DNA barcoding, as a reliable, cost-effective, and accessible solution for species identification, has been widely used [15,45–47]. The CBOL Plant Working Group proposed the combination of *rbcL* + *matK* as a plant barcode [48]. The two fragments were characterized by good primer universality, high amplification efficiency, good sequence quality, and high discrimination power. Numerous studies have revealed that *rbcL* and *matK* are informative for the resolution of phylogenetic issues at higher taxonomic levels, but are not useful for dealing with problems at lower levels, such as species discrimination, because these regions often lack variations in closely related species, especially those that have diverged recently in evolution [49]. The low success rate of *matK* amplification has also been observed by other researchers [50] and was confirmed by our results. The noncoding plastid marker *trnL-F* has been researched as a DNA barcode for land plants in general [51] and for bryophytes (mosses) [52]. This marker also had a good identification success rate in the present study. De Groot Ga et al. (2011) used *rbcL* and *trnL-F* as two-loci DNA barcodes for the identification of NW-European ferns, and based on the combined *rbcL* and *trnL-F* data set, all genera and all species with non-equal chloroplast genomes formed their own well-supported monophyletic clade, which indicated the high discriminatory power of these loci [53]. These findings agreed with our results.

According to our results, the method of combining morphology and DNA barcoding could be reliable, cost-effective, and accessible for species identification.

## Conclusions

Based on the above analyses, *D*. *simulans* is widely distributed in Yunnan Province and Hainan Island in China, and also in tropical Asia, specifically Indonesia. This species should be considered an independent species that is sister to *D*. *cantonense* based on obvious difference in morphological traits and the genetic gaps. According to the International Code of Nomenclature for algae, fungi, and plants (Melbourne Code) [54], the species *Diplopterygium yunnanense* (Ching) Ching ex X. C. Zhang that is treated as a synonymy of *D*. *giganteum* should be treated as the synonymy of *D*. *simulans* which was published previously.

We should pay close attention to rechecking synonymy to find “new” species, which may be an important factor in finding biodiversity in the future, although there was only one species treated in this study.

## Supporting information

S1 Table Information about the specimens used in the present study. Voucher number, species, collection site and GenBank accession numbers of the *rbcL*, *matK*, *trnL-F*, *atpB* and *rps4* sequences utilized for this study.
(DOCX)

S2 Table PCR primers used in the present study. DNA regions, primer name, sequence (5’~3’) and source references of the primers utilized for this study.
(DOCX)

S3 Table PCR reaction conditions used in this study. The conditions of PCR reaction (pre degeneration, degeneration, annealing, extension and termination of the extension) for five loci (*rbcL*, *matK*, *trnL-F*, *atpB* and *rps4*) utilized for this study.
(DOCX)
