# Supplementary material for: Re-establishment of species from synonymies based on DNA barcoding and phylogenetic analysis using Diplopterygium simulans (Gleicheniaceae) as an example
Source: PLoS One. 2017 Mar 15;12(3):e0164604. doi: 10.1371/journal.pone.0164604 (PMC5351838; doi:10.1371/journal.pone.0164604)
Supplement: S1 Table — Voucher number, species, collection site and GenBank accession numbers of the rbcL, matK, trnL-F, atpB and rps4 sequences utilized for this study. (DOCX) [file pone.0164604.s001.docx]

**S1 Table: Information about the specimens used in the present study.**

| Voucher number | Species | Collection site | GenBank Accession Number | | | | | | |
| --- | --- | --- | --- | --- | --- | --- | --- | --- | --- |
|  |  |  | *rbcL* | *matK* | *trnL-F* | *atpB* | | | *rps4* |
| LHM1998 | *Diplopterygium glaucum* | Guangdong, China | KU936572 | KU936507 | KU936702 | KU877738 | KU936637 | | |
| MES037 | *Dip. glaucum* | Guangxi, China | KU936573 | KU936508 | KU936703 | KU877739 | KU936638 | | |
| MS135 | *Dip. glaucum* | Hunan, China | KU936574 | KU936509 | KU936704 | KU877740 | KU936639 | | |
| SG048 | *Dip. glaucum* | Fujian, China | KU936575 | KU936510 | KU936705 | KU877741 | KU936640 | | |
| SG152 | *Dip. glaucum* | Fujian, China | KU936576 | KU936511 | KU936706 | KU877742 | KU936641 | | |
| YYH12652 | *Dip. glaucum* | Hunan, China | KU936577 | KU936512 | KU936707 | KU877743 | KU936642 | | |
| YYH13220 | *Dip. glaucum* | Hunan, China | KU936578 | KU936513 | KU936708 | KU877744 | KU936643 | | |
| YYH13637 | *Dip. glaucum* | Guangxi, China | KU936579 | KU936514 | KU936709 | KU877745 | KU936644 | | |
| YYH13896 | *Dip. glaucum* | Sichuan, China | KU936580 | KU936515 | KU936710 | KU877746 | KU936645 | | |
| YYH13928 | *Dip. glaucum* | Sichuan, China | KU936581 | KU936516 | KU936711 | KU877747 | KU936646 | | |
| ZXL2013712007 | *Dip. glaucum* | Hubei, China | KU936582 | KU936517 | KU936712 | KU877748 | KU936647 | | |
| ZXL5327 | *Dip. glaucum* | Zhejiang, China | KU936583 | KU936518 | KU936713 | KU877749 | KU936648 | | |
| HNS055 | *Dip. blotianum* | Hainan, China | KU936584 | KU936519 | KU936714 | KU877750 | KU936649 | | |
| HNS083 | *Dip. blotianum* | Hainan, China | KU936585 | KU936520 | KU936715 | KU877751 | KU936650 | | |
| LHM1900 | *Dip. blotianum* | Guangdong, China | KU936586 | KU936521 | KU936716 | KU877752 | KU936651 | | |
| XP607 | *Dip. blotianum* | Hainan, China | KU936587 | KU936522 | KU936717 | KU877753 | KU936652 | | |
| YYH12340 | *Dip. blotianum* | Yunnan, China | KU936588 | KU936523 | KU936718 | KU877754 | KU936653 | | |
| YYH13160 | *Dip. blotianum* | Hong Kong, China | KU936589 | KU936524 | KU936719 | KU877755 | KU936654 | | |
| YYH13204A | *Dip. blotianum* | Hainan, China | KU936590 | KU936525 | KU936720 | KU877756 | KU936655 | | |
| YYH13458 | *Dip. blotianum* | Guangxi, China | KU936591 | KU936526 | KU936721 | KU877757 | KU936656 | | |
| YYH13478 | *Dip. blotianum* | Guangxi, China | KU936592 | KU936527 | KU936722 | KU877758 | KU936657 | | |
| YYH13676 | *Dip. blotianum* | Guangdong, China | KU936593 | KU936528 | KU936723 | KU877759 | KU936658 | | |
| GBJ02943 | *Dip. chinense* | Fujian, China | KU936594 | KU936529 | KU936724 | KU877760 | KU936659 | | |
| GBJ02978 | *Dip. chinense* | Fujian, China | KU936595 | KU936530 | KU936725 | KU877761 | KU936660 | | |
| LHM1929 | *Dip. chinense* | Guangdong, China | KU936596 | KU936531 | KU936726 | KU877762 | KU936661 | | |
| LHM2010 | *Dip. chinense* | Guangdong, China | KU936597 | KU936532 | KU936727 | KU877763 | KU936662 | | |
| MS107 | *Dip. chinense* | Hunan, China | KU936598 | KU936533 | KU936728 | KU877764 | KU936663 | | |
| WZS001 | *Dip. chinense* | Hainan, China | KU936599 | KU936534 | KU936729 | KU877765 | KU936664 | | |
| YYH12637 | *Dip. chinense* | Guangdong, China | KU936600 | KU936535 | KU936730 | KU877766 | KU936665 | | |
| YYH13203 | *Dip. chinense* | Hainan, China | KU936601 | KU936536 | KU936731 | KU877767 | KU936666 | | |
| YYH13255_1 | *Dip. chinense* | Guangxi, China | KU936602 | KU936537 | KU936732 | KU877768 | KU936667 | | |
| YYH13255_2 | *Dip. chinense* | Guangxi, China | KU936603 | KU936538 | KU936733 | KU877769 | KU936668 | | |
| YYH13255_3 | *Dip. chinense* | Guangxi, China | KU936604 | KU936539 | KU936734 | KU877770 | KU936669 | | |
| YYH13816 | *Dip. chinense* | Sichuan, China | KU936605 | KU936540 | KU936735 | KU877771 | KU936670 | | |
| HNS057 | *Dip. cantonense* | Hainan, China | KU936606 | KU936541 | KU936736 | KU877772 | KU936671 | | |
| HNS064 | *Dip. cantonense* | Hainan, China | KU936607 | KU936542 | KU936737 | KU877773 | KU936672 | | |
| HNS073 | *Dip. cantonense* | Hainan, China | KU936608 | KU936543 | KU936738 | KU877774 | KU936673 | | |
| HNS075 | *Dip. cantonense* | Hainan, China | KU936609 | KU936544 | KU936739 | KU877775 | KU936674 | | |
| HNS076 | *Dip. cantonense* | Hainan, China | KU936610 | KU936545 | KU936740 | KU877776 | KU936675 | | |
| HNS078 | *Dip. cantonense* | Hainan, China | KU936611 | KU936546 | KU936741 | KU877777 | KU936676 | | |
| LHM1908 | *Dip. cantonense* | Guangdong, China | KU936612 | KU936547 | KU936742 | KU877778 | KU936677 | | |
| Voucher number | **Species** | **Collection site** | GenBank Accession Number | | | | | | |
|  |  |  | *rbcL* | *matK* | *trnL-F* | *atpB* | | *rps4* | |
| YYH12435 | *Dip. cantonense* | Guangdong, China | KU936613 | KU936548 | KU936743 | KU877779 | KU936678 | | |
| YYH13192 | *Dip. cantonense* | Hainan, China | KU936614 | KU936549 | KU936744 | KU877780 | KU936679 | | |
| YYH13199 | *Dip. cantonense* | Hainan, China | KU936615 | KU936550 | KU936745 | KU877781 | KU936680 | | |
| BLD057 | *Dip. simulans* | Bali, Indonesia | KU936616 | KU936551 | KU936746 | KU877782 | KU936681 | | |
| HNS058 | *Dip. simulans* | Hainan, China | KU936617 | KU936552 | KU936747 | KU877783 | KU936682 | | |
| HNS060 | *Dip. simulans* | Hainan, China | KU936618 | KU936553 | KU936748 | KU877784 | KU936683 | | |
| HNS062 | *Dip. simulans* | Hainan, China | KU936619 | KU936554 | KU936749 | KU877785 | KU936684 | | |
| HNS086 | *Dip. simulans* | Hainan, China | KU936620 | KU936555 | KU936750 | KU877786 | KU936685 | | |
| YYH12261 | *Dip. yunnanense* | Yunnan, China | KU936621 | KU936556 | KU936751 | KU877787 | KU936686 | | |
| YYH12339 | *Dip. yunnanense* | Yunnan, China | KU936622 | KU936557 | KU936752 | KU877788 | KU936687 | | |
| YYH13198 | *Dip. simulans* | Hainan, China | KU936623 | KU936558 | KU936753 | KU877789 | KU936688 | | |
| YYH13200 | *Dip. simulans* | Hainan, China | KU936624 | KU936559 | KU936754 | KU877790 | KU936689 | | |
| YYH12139 | *Dip. giganteum* | Yunnan, China | KU936625 | KU936560 | KU936755 | KU877791 | KU936690 | | |
| YYH12149 | *Dip. giganteum* | Yunnan, China | KU936626 | KU936561 | KU936756 | KU877792 | KU936691 | | |
| YYH13917 | *Dip. maximum* | Sichuan, China | KU936627 | KU936562 | KU936757 | KU877793 | KU936692 | | |
| ZXL201379001 | *Dip. maximum* | Chongqing, China | KU936628 | KU936563 | KU936758 | KU877794 | KU936693 | | |
| MES028 | *Dip. laevissimum* | Guangxi, China | KU936629 | KU936564 | KU936759 | KU877795 | KU936694 | | |
| MS035 | *Dip. laevissimum* | Hunan, China | KU936630 | KU936565 | KU936760 | KU877796 | KU936695 | | |
| YYH13221 | *Dip. laevissimum* | Hunan, China | KU936631 | KU936566 | KU936761 | KU877797 | KU936696 | | |
| YYH13276 | *Dip. laevissimum* | Guangxi, China | KU936632 | KU936567 | KU936762 | KU877798 | KU936697 | | |
| ZXL10017 | *Dip. laevissimum* | Guangdong, China | KU936633 | KU936568 | KU936763 | KU877799 | KU936698 | | |
| YYH13448 | *Dicranopteris linearis* | Guangxi, China | KU936634 | KU936569 | KU936764 | KU877800 | KU936699 | | |
| ZXL5219 | *Sticherus laevigatus* | Guangdong, China | KU936635 | KU936570 | KU936765 | KU877801 | KU936700 | | |
| YYH11631 | *Dip. blotanum* | Taiwan, China | KU936636 | KU936571 | KU936766 | KU936767 | KU936701 | | |
