# Supplementary material for: Re-establishment of species from synonymies based on DNA barcoding and phylogenetic analysis using Diplopterygium simulans (Gleicheniaceae) as an example
Source: PLoS One. 2017 Mar 15;12(3):e0164604. doi: 10.1371/journal.pone.0164604 (PMC5351838; doi:10.1371/journal.pone.0164604)
Supplement: S2 Table — DNA regions, primer name, sequence (5’~3’) and source references of the primers utilized for this study. (DOCX) [file pone.0164604.s002.docx]

**S2 Table. PCR primers used in the present study.**

| DNA regions | Primer name | Sequence(5’~3’) | Reference |
| --- | --- | --- | --- |
| *rbcL* | F1  R1379 | ATGTCACCACAAACGGAGAC  GCAGCTAATTCAGGACTCC | Li et al., 2004 [1] |
| *atpB* | F  R | AGCTTCATCGATGTTACC  GTTGGTGAAACTACTCTTGG | Li et al., 2010 [2] |
| *rps4* | F  R | ATGTCCCGTTATCGAGGACC  GGAATGATACTCGACGACTAG | Li et al., 2010 [2] |
| *trnL-F* | e  f | ATTTGAACTGGTGACACGAG  GGTTCAAGTCCCTCTATCCC | Small et al., 2005 [3] |
| *matK* | *matK*rAGK  *matK*fEDR | CGTRTTGTACTYYTRTGTTTRCVAGC  ATTCATTCRATRTTTTTATTTHTGGARGAYAGATT | Kuo et al., 2011 [4] |

Reference：

1. Li, C., Lu, S., & Yang, Q. (2004). Asian origin for Polystichum (Dryopteridaceae) based on rbcL sequences. *Chinese Science Bulletin, 49*(11), 1146-1150.
2. Li, C., Lu, S., Ma, J., & Yang, Q. (2010). Phylogeny and divergence of gleicheniaceae inferred from three plastid genes. *Acta Palaeontologica Sinica, 49*(1), 64-72.
3. Small, R. L., Lickey, E. B., Shaw, J., & Hauk, W. D. (2005). Amplification of noncoding chloroplast DNA for phylogenetic studies in lycophytes and monilophytes with a comparative example of relative phylogenetic utility from Ophioglossaceae. *Molecular Phylogenetics and Evolution, 36*(3), 509-522.
4. Kuo, L.-Y., Li, F.-W., Chiou, W.-L., & Wang, C.-N. (2011). First insights into fern matK phylogeny. *Molecular Phylogenetics and Evolution, 59*(3), 556-566.
