# Supplementary material for: Re-establishment of species from synonymies based on DNA barcoding and phylogenetic analysis using Diplopterygium simulans (Gleicheniaceae) as an example
Source: PLoS One. 2017 Mar 15;12(3):e0164604. doi: 10.1371/journal.pone.0164604 (PMC5351838; doi:10.1371/journal.pone.0164604)
Supplement: S3 Table — The conditions of PCR reaction (pre degeneration, degeneration, annealing, extension and termination of the extension) for five loci (rbcL, matK, trnL-F, atpB and rps4) utilized for this study. (DOCX) [file pone.0164604.s003.docx]

**S3 Table. PCR reaction conditions used in this study.**

|  | *rbcL* | *atpB* | *rps4* | *trnL-F* | *matK* |
| --- | --- | --- | --- | --- | --- |
| Pre degeneration | 94℃,5min | 94℃,5min | 94℃,5min | 94℃,3min | 95℃,5min |
| Degeneration | 94℃,0.5min | 94℃,0.5min | 94℃,0.5min | 94℃,0.5min | 94℃,0.5min |
| Annealing | 50℃,1min | 52℃,1min | 56℃,1min | 52℃,1min | 50℃,0.5min |
| Extension | 72℃,1.5min | 72℃,1.2min | 72℃,0.5min | 72℃,1min | 72℃,1min |
| Termination of the extension | 72℃,7min | 72℃,7min | 72℃,7min | 72℃,10min | 72℃,7min |
